# Supplementary material for: Accelerated fetal growth velocity across the third trimester is associated with increased shoulder dystocia risk among fetuses who are not large-for-gestational-age: A prospective observational cohort study
Source: PLoS One. 2021 Oct 20;16(10):e0258634. doi: 10.1371/journal.pone.0258634 (PMC8528331; doi:10.1371/journal.pone.0258634)
Supplement: Attachment — Submitted filename: Response to reviewers.docx [file pone.0258634.s002.docx]

Dear Editors of PLOS ONE,

**Re:	Manuscript PONE-D-21-00919**

**MacDonald *et al*, ‘Accelerated fetal growth velocity across the third trimester is associated with increased shoulder dystocia risk among fetuses who are not large-for-gestational-age: A prospective observational cohort study’**

We thank the reviewers for their feedback and suggestions. Here we are pleased to provide our responses, and submit this revised manuscript for further consideration. For ease of review, we have tracked changes in one of the versions of the manuscript – and have referenced the line numbers of the changes in the tracked changes version in this response letter.

We look forward to your further correspondence,

Dr Teresa MacDonald, corresponding author.

**REVIEWER #1**

**MacDonald et al present an interesting cohort study on the risk of shoulder dystocia according to accelerated fetal growth.**

We are glad to read that the reviewer found our study interesting. Thank you for this comment.

**I have the following comments/questions:
1. Methods
Line 34: “We then compared the predictive 35 performances of accelerated EFW and AC growth velocities (>30 centiles gained) with 36-week 36 EFW >95th centile for shoulder dystocia among the cohort planned for vaginal birth”. This sentence is not clear. The authors’ analysis consisted in 2 steps, could they clarify them?**

In light of this comment we have added to the methods explained in the abstract, so that it now reads as follows (Abstract, Methods subsection, Page 2, lines 32-38):

"We examined the odds of shoulder dystocia with increasing EFW and AC growth velocities among women with 36-week EFW ≤95th centile (non-LGA), who went on to have a vaginal birth. We then examined the relative risk (RR) of shoulder dystocia in cases of accelerated EFW and AC growth velocities (>30 centiles gained). Finally, we compared the predictive performances of accelerated fetal growth velocities to 36-week EFW >95th centile for shoulder dystocia among the cohort planned for vaginal birth."

**2**. **Results
Why did the authors use both OR and RR to describe their results?**

We believe that it is statistically correct to present the results of our logistic regression analysis as an odds ratio. Here, the odds of a dichotomous outcome - shoulder dystocia or not - are increased by each centile increase in growth velocity (a continuous variable). The statistics associated with this analysis are truly best represented as odds ratios and hence that is what we have presented.

In contrast, we present relative risks when the cohort is dichotomised according to accelerated growth velocity or not. This is when we present the occurrence of a dichotomous outcome according to a dichotomous risk categorisation. Here we have actively chosen to present relative risk as we believe this information is best understood by clinicians. In essence, we are attempting to present clinically relevant cut-offs, and a quantification of how great an increase in centiles increases the risk of shoulder dystocia. We believe presenting this information as a relative risk improves the potential for clinical translation of the data. Further, presenting a relative risk after Fisher's exact of Chi Square testing is statistically sound.

Moreover, presenting the data in this way is also consistent with our previous publications on reduced fetal growth velocities [1, 2], and it is interesting that a magnitude of 30 centiles difference seems to be a clinically relevant cut-off for both reducing or accelerating fetal growth velocity.

**3. Introduction
The authors should mention the prevalence of shoulder dystocia**

The second sentence of the Introduction (page 4, lines 59-62) has now been adjusted to include this information. It now reads as:

"Shoulder dystocia complicates between 0.2% and 3.0% of all vaginal deliveries [6] and is associated with brachial plexus injury, fractures, and birth asphyxia for the infant; and increased maternal haemorrhage and genital tract trauma [5, 7]."

**4. The following quote does not seem to be appropriate for an international audience: line 66 “consistently demonstrating poor positive predictive value [1, 5]. That clinicians require better ways of identifying women at risk of shoulder dystocia is evidenced by the high profile case successfully lodged against the Lanarkshire Health Board after Nadine Montgomery’s son suffered cerebral palsy following shoulder dystocia”**

In light of the comments received from both reviewers, reference to the Montgomery case in the Introduction has now been deleted. The Discussion also referenced the case and this has now been altered slightly given that the introduction to this case is no longer present.

The relevant section of the Discussion (page 17, lines 301-303) now reads as:

“Even a landmark legal case [10] has highlighted that, given the potential severe consequences of shoulder dystocia, the onus is on clinicians to provide pregnant women with risk assessment and options regarding mode of birth.”

**5. Line 79 “Importantly, the corollary was also noted: the higher the fetal growth velocity, the higher the neonatal fat stores” Was this demonstrated in the authors’ previous publication?**

Yes, this finding is the result of our linear regression analysis comparing the correlations between fetal growth velocity and neonatal fat stores - measured as PEA POD body fat percentage, skinfold body fat percentage and Ponderal Index.

To make this more clear to the reader, the sentence has now been adjusted to read as follows, and with specific further reference to the same publication (page 5, lines 81-83):

"Specifically, we discovered that the lower the fetal growth velocity, the lower the neonatal fat stores [11]. This means that the corollary also holds true: the higher the fetal growth velocity, the higher the neonatal fat stores [11]."

**6. What do the authors mean by “pathological overgrowth” (line 84)? A fat distribution that mimics diabetes mellitus, increasing the risk of shoulder dystocia independently of the actual birthweight?**

We have added to the sentence to add clarity to what we mean by pathological overgrowth, so that it now reads as (Introduction, page 5, lines 85-88):

"We hypothesised that if reduced fetal growth velocity represents relative placental insufficiency (growth less than expected by genetic potential) among fetuses that are not small [11], accelerated fetal growth velocity might indicate pathological overgrowth (growth in excess of the genetic growth potential) even where the EFW is ≤95th centile."

**7. Methods/Results
Line 165 “We assessed the relationships between EFW and AC growth velocities and shoulder dystocia in two ways: (i) we analysed the growth velocities as continuous variables against the outcome of interest – shoulder dystocia – using logistic regression. This determined the odds of shoulder dystocia per centile increase in 28-36 week EFW or AC growth velocity; and (ii) we assessed our pre-defined dichotomous thresholds, increase of EFW and AC growth velocities of >30 centiles over eight weeks compared to the remainder of the cohort, using Fisher’s exact test. This ascertained the relative risk (RR) of shoulder dystocia where an increase in centile of this magnitude was seen. Finally we compared the predictive performances of accelerated 28-36 week EFW and AC growth velocities (>30 centiles) and 36-week EFW >95th centile for shoulder dystocia. These performance parameters were calculated from all participants planned for vaginal delivery – replicating the cohort at potential risk of shoulder dystocia when seen in the antenatal clinic”**

**The statistical approach to data analysis is confusing. The authors intend to study the risk of shoulder dystocia according to growth velocity among fetuses with EFW < 95%ile. Therefore, they should present a univariate analysis where the baseline characteristics of the women enrolled are analyzed according to presence or absence of shoulder dystocia at birth, among these characteristics they should also describe growth velocity, both as a continuous and as a categorical variable. Then, they should build a multivariate regression model studying the relationship between the dependent variable (shoulder dystocia) and the dependent variable growth velocity (either continuous or categorical), controlling for confounding**

We thank the reviewer for bringing this error to our attention. We have now reanalysed our data as the reviewer has suggested. Table 1 now reports maternal characteristics and delivery outcomes for cases of shoulder dystocia compared to the rest of the cohort, and includes comparisons between the groups of the 28-36 week EFW and AC velocities, and rates of accelerated 28-36 week EFW and AC velocities being observed.

This new comparison found that maternal age and booking BMI were significantly different between the two groups, and as such, we performed our multivariate logistic regression again, accounting for these two potential confounders. As such, new ORs are reported for the odds of shoulder dystocia for each centile increase in EFW or AC over the 8 weeks. These remain significantly elevated – with odds that are slightly higher than we previously reported. Thus, the main findings of our study remain unchanged. The new results are all reported in the Results section (pages 10-13) of the manuscript and the abstract has also been updated to reflect the new odds ratios and corresponding p values.

**8. Can the authors comment on the prevalence of shoulder dystocia, as it appears to be high for a population that does not include only diabetic mothers ?**

The prevalence of shoulder dystocia in our population is addressed in the Discussion when we state (page 20, lines 360-365):

"Strengths of this study include that it was prospective and blinded, minimising the risk of intervention bias, and that we applied a robust and clinically significant definition of shoulder dystocia. We specifically did not include a time-based definition of >60 seconds between delivery of the infant's head and body as this potentially defines over 10% of vaginal births as complicated by shoulder dystocia [6, 24]. Instead, our shoulder dystocia rates (2.0% of pregnancies and 2.8% of vaginal births) are in keeping with its reported incidence [6]."

We have now added to this statement further (Discussion, page 20, lines 365-369), saying:

"That our prevalence is at the higher end of the range may reflect the high quality of our obstetrics trainees and midwives, our regular simulation training which includes shoulder dystocia emergencies, and a culture of early recognition and recourse to emergency manoeuvres, as well as calling for help and of clear documentation."

**REVIEWER #2**

**This conceptually interesting paper by McDonald and peers examines a data set on fetal growth and the outcome of shoulder social. Specifically, this manuscript concentrates on the growth velocity of the child, which is an interesting novel marker. The paper is generally well written and the statistical approach is suitable for such an analysis. The following comments are meant to strengthen the paper:**

We thank the reviewer for this positive feedback on our work.

**1. The largest and potentially fatal flaw in this paper is the sample size which included 347 individuals. Though they are able to achieve statistical significance in their primary outcome this is not reassuring as it based on 6 cases of shoulder dystocia. If published the conclusion should be worded more skeptically and the need for a larger population strongly emphasized. Please note that I remain intrigued by the potential of this marker but concerned about its implementation based on the limited number of cases.**

We agree with the reviewer on this point and have specifically discussed our sample size as a limitation to the study in the Discussion where we state (page 20, lines 376-378:

"Secondly, sample size is the main limitation of these findings. That our study was small, with only seven cases of shoulder dystocia, means it requires validation in a larger cohort"

We have now added to this, stating further in the Discussion (page 21, lines 384-386):

"However, given our small number of cases, the need for validation in larger cohorts cannot be overemphasised and we would caution against clinical implementation at this stage where our findings are based on such a limited number of cases."

And we have added to the Conclusion to state (page 22, lines 390-391):

"This finding warrants urgent further validation in a much larger cohort."

**2. Though the occurrence of shoulder dystocia was objectively assessed, the clinical meaninfullness of this outcome is unclear. No comments about whether the shoulder dystocia resulted in maternal or fetal harm is discussed. Outcomes related to BPI, HIE, and maternal pelvic trauma/hemorrhage should be added.**

As the reviewer correctly points out, our sample size was only just large enough to assess for the increased risk of shoulder dystocia that we have described. Our cohort of only 7 shoulder dystocia cases was too small in which to further analyse for subsequent consequences of shoulder dystocia. However, in light of this comment we have added the following line to our discussion of limitations so that it now reads (page 20, lines 377-380):

"That our study was small, with only seven cases of shoulder dystocia, means it requires validation in a larger cohort in order to assess for important sequelae of shoulder dystocia such as brachial plexus injury, hypoxic ischaemic encephalopathy, or maternal anal sphincter injury or haemorrhage."

**3. Though the test performance is reported the challenge is that to avoid the shoulder dystocia significant numbers of cesareans would need to be performed (8 at 12.5% and 14 at 7.1%) to prevent one shoulder dystocia that may not result in harm as discussed in point 2. For balance the maternal risks of this should be discussed.**

We concur that unintended consequences of assessments involving excessive fetal growth have traditionally focussed on the potential for excess unnecessary caesarean sections. However, throughout this manuscript we have attempted to reframe this in light of recent evidence regarding the safety and efficacy of early induction of labour to reduce the risk – and consequences – of shoulder dystocia. Hence, we discuss throughout the paper that should better predictors of shoulder dystocia be identified, this might reduce shoulder dystocia through induction of labour, timed earlier at term.

This is explained in the Introduction when we state (page 4, lines 62-65):

“…clinicians have an opportunity to intervene and reduce these risks. A recent systematic review has reported that induction of labour in cases of suspected macrosomia (estimated fetal weight (EFW) >95^th^ centile [8]) reduces both shoulder dystocia and fracture risk, compared to expectant management [9].”

We again allude to induction of labour as a potential clinical intervention to reduce shoulder dystocia (and not to elective caesarean section) in the Discussion (page 17, lines 294-298) when we state:

“Further, in our cohort, accelerated growth velocities demonstrate better predictive performance for shoulder dystocia than EFW >95^th^ centile – a threshold currently used to recommend earlier term induction [8, 9]. Accelerated fetal growth velocity could thus potentially alert clinicians of otherwise unsuspected increased shoulder dystocia risk, improving the prediction, and prevention, of this serious obstetric emergency.”

And also where the Discussion (page 17, lines 303-309 includes the following:

“Evidence is accumulating for the safety and efficacy of early term induction to reduce shoulder dystocia risk [9] which may be preferred by clinicians and women to elective caesarean given ultrasound has poorer accuracy, and higher false positive rates, in large babies [14-16]. Induction is not associated with a significant increase in emergency caesarean section [9]. Induction of labour for LGA fetuses (clinical suspicion followed by EFW >95^th^ centile), represents the only risk factor and intervention dyad shown in a randomised controlled trial to reduce shoulder dystocia [8].”

**4. The term used of “Emergency cesarean” in table 1 & 4 I believe is better conveyed as unscheduled**

“Emergency caesarean” has been changed to “Unscheduled caesarean” in Table 4 (now become Table 3; page 15), and in the text related to it, as suggested by the reviewer.

**5. Table 2 & 3 should be combined or woven into the text. There is not enough information to warrant a separate table.**

The data presented in Table 2 was already described in full in the text of the manuscript, and as such, Table 2 has been deleted. Table 3 has been kept in the manuscript, (relabelled as Table 2), as it highlights the main clinically relevant finding of the paper to potential readers. We feel it is to the paper’s advantage to highlight this result in a table rather than reporting it in the text alone.

**6. The reference provided to a law suit in the UK (line 66) is fear mongering and not appropriate to include.**

In light of the comments received from both reviewers, reference to the Montgomery case in the Introduction has now been deleted. The discussion also referenced the case and this has now been altered slightly given that the introduction to this case is no longer present.

The relevant section of the Discussion (page 17, lines 301-303) now reads as:

“Even a landmark legal case [10] has highlighted that, given the potential severe consequences of shoulder dystocia, the onus is on clinicians to provide pregnant women with risk assessment and options regarding mode of birth.”

***References in this response letter (see manuscript for references listed in quoted sections)***

*1.	MacDonald TM, Hui L, Tong S, Robinson AJ, Dane KM, Middleton AL, et al. Reduced growth velocity across the third trimester is associated with placental insufficiency in fetuses born at a normal birthweight: a prospective cohort study. BMC Med. 2017;15(1):164. Epub 2017/09/01. doi: 10.1186/s12916-017-0928-z. PubMed PMID: 28854913; PubMed Central PMCID: PMCPMC5577811.*

*2.	Kennedy LM, Tong S, Robinson AJ, Hiscock RJ, Hui L, Dane KM, et al. Reduced growth velocity from the mid-trimester is associated with placental insufficiency in fetuses born at a normal birthweight. BMC Med. 2020;18(1):395. Epub 2020/12/29. doi: 10.1186/s12916-020-01869-3. PubMed PMID: 33357243; PubMed Central PMCID: PMCPMC7758928.*
